# Dual Tumor Pathogenesis in the Gastrointestinal Tract: Synchronous Rectal Schwannoma and Gallbladder Papillary Adenocarcinoma—A Case Report

**DOI:** 10.3390/reports9010014

**Published:** 2025-12-31

**Authors:** Adrian Cotovanu, Catalin Dumitru Cosma, Calin Molnar, Simona Gurzu, Marius-Alexandru Beleaua, Vlad Olimpiu Butiurca, Marian Botoncea

**Affiliations:** 1 Department of Surgery, Faculty of Medicine, George Emil Palade University of Medicine, Pharmacy, Science and Technology of Târgu Mureș, 540139 Târgu Mureș, Romaniasimonagurzu@yahoo.com (S.G.); beleaua.marius@umfst.ro (M.-A.B.);; 2 General Surgery Clinic No. 1, County Emergency Clinical Hospital of Târgu-Mureș, 540136 Târgu-Mureș, Romania; 3 Department of Pathology, Faculty of Medicine, George Emil Palade University of Medicine, Pharmacy, Science and Technology of Târgu Mureș, 540139 Târgu Mureș, Romania

**Keywords:** synchronous tumors, rectal schwannoma, gallbladder papillary adenocarcinoma, benign–malignant coexistence, gastrointestinal surgery, dual tumor pathogenesis, case-report

## Abstract

**Background and Clinical Significance**: Synchronous gastrointestinal tumors are exceptionally rare, particularly when combining histologically distinct benign and malignant components. Schwannomas represent uncommon mesenchymal tumors of the gastrointestinal tract, most frequently arising in the stomach, while rectal localization is exceedingly unusual. Papillary adenocarcinoma of the gallbladder is an aggressive malignant entity derived from intracholecystic papillary–tubular neoplasms (ICPNs). The coexistence of these two unrelated neoplasms has not been previously reported, making this case of dual tumor pathogenesis clinically and academically significant. **Case Presentation**: A 68-year-old female was admitted for surgical management of grade IV uterovaginal prolapse. Preoperative imaging incidentally revealed a well-circumscribed rectal wall mass and gallstones. A combined abdominopelvic operation was performed, including total hysterectomy with bilateral adnexectomy (Wiart procedure), rectosigmoid resection with colorectal anastomosis, and bipolar cholecystectomy. Intraoperatively, a firm intramural rectal lesion and a friable papillary mass in the gallbladder fundus were identified. Histopathologic examination confirmed a benign rectal schwannoma (S-100 positive, CD117/DOG-1 negative) and a papillary adenocarcinoma of the gallbladder, pT3N0M0, with clear resection margins and no lymphovascular or perineural invasion. The postoperative course was uneventful, and the patient remained disease-free at six-month follow-up. **Conclusions**: This case represents an exceedingly rare benign–malignant synchronous tumor association. The simultaneous occurrence of rectal schwannoma and gallbladder papillary adenocarcinoma underscores the importance of thorough intraoperative exploration and histopathologic evaluation. Complete resection with negative margins and multidisciplinary follow-up remains crucial for optimal outcomes and contributes to understanding dual tumor pathogenesis within the gastrointestinal tract.

## 1. Introduction and Clinical Significance

Synchronous neoplastic lesions within the gastrointestinal tract represent a rare but clinically significant occurrence, particularly when they involve histologically distinct benign and malignant components. Schwannomas are benign mesenchymal neoplasms that account for less than 5% of all mesenchymal tumors of the gastrointestinal tract [1]. Their occurrence in the colon and rectum is exceptional, with only isolated cases described in the literature [2,3]. Typically, these tumors manifest as submucosal lesions with spindle-cell morphology and an immunohistochemical profile characterized by S-100 protein positivity and negativity for CD117, DOG-1, and desmin [4,5,6]. Complete surgical excision remains the treatment of choice and is associated with excellent long-term outcomes.

In contrast, gallbladder carcinoma is an uncommon malignancy, frequently diagnosed at advanced stages owing to its nonspecific clinical presentation [7,8]. Among its histologic subtypes, papillary adenocarcinoma—often arising from intracholecystic papillary–tubular neoplasms (ICPNs)—demonstrates distinctive morphologic and biologic behavior, with comparatively better resectability and prognosis than conventional infiltrative adenocarcinoma [9,10]. Despite these favorable characteristics, accurate preoperative diagnosis remains challenging, as imaging findings often overlap with those of benign cholelithiasis or adenomyomatous hyperplasia [11].

The coexistence of rectal schwannoma and gallbladder papillary adenocarcinoma is exceedingly rare, with no prior cases documented to our knowledge. Reports of synchronous gastrointestinal tumors typically involve double primary malignancies, such as combined hepatocellular and gallbladder carcinoma or concurrent colonic and gallbladder adenocarcinoma [12,13,14]. The underlying mechanisms remain speculative, encompassing coincidental development due to age-related genetic instability, shared carcinogenic exposures, or molecular predispositions promoting multiorgan tumorigenesis. Such occurrences highlight the concept of dual tumor pathogenesis, where distinct neoplasms may arise synchronously within separate organ systems through either independent or interconnected biological pathways [15].

This report presents a unique case of synchronous benign rectal schwannoma and malignant papillary adenocarcinoma of the gallbladder identified incidentally during surgery for genital prolapse. The synchronous occurrence of a benign mesenchymal tumor and a malignant epithelial neoplasm within the gastrointestinal tract is exceedingly rare, and its recognition is clinically relevant for diagnostic accuracy, surgical strategy, and postoperative surveillance.

## 2. Case Presentation

**Figure 1:**
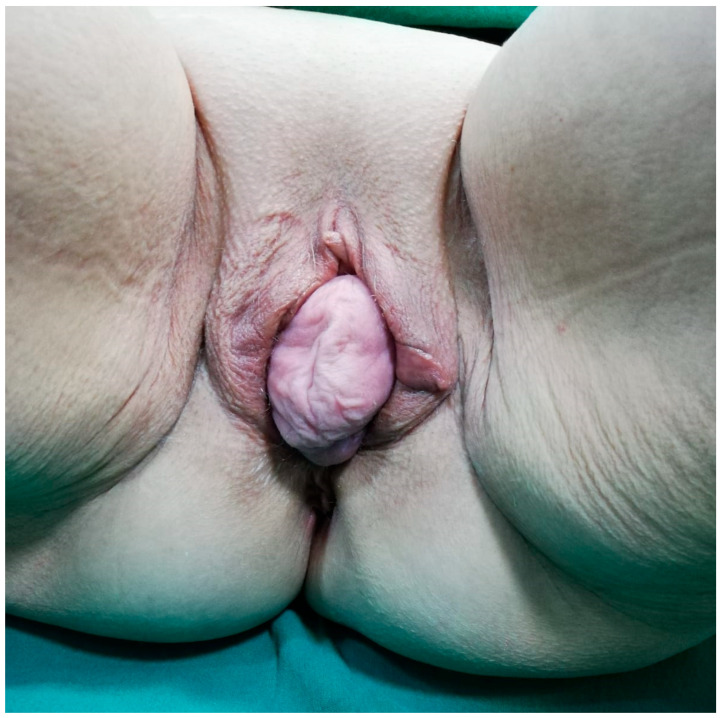
Preoperative view showing complete uterovaginal procidentia (POP-Q stage IV) with externalized cervix and associated perineal descent, the primary reason for the patient’s admission.

A 68-year-old female patient received a referral to the Department of General Surgery I at Emergency County Hospital Târgu Mureș for surgical treatment of her severe genital prolapse (POP-Q stage IV) and her ongoing constipation and occasional rectal pain. The patient showed no symptoms during the examination because he did not experience rectal bleeding or weight loss, or changes in stool thickness. The patient had mild hypertension, which she managed with amlodipine, and she had a past diagnosis of cholelithiasis but never experienced biliary colic. She was a nonsmoker, had no history of alcohol consumption, and reported no familial cancer syndromes or prior malignancies (Figure 1).

**Figure 2:**
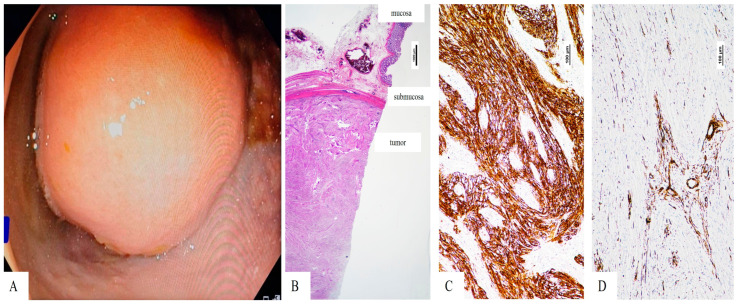
Rectalschwannoma—(**A**). Colonoscopic view of the intraluminal, smooth-surfaced rectal lesion located 8 cm from the anal verge, causing partial luminal stenosis—(**B**). Microscopic view of the submucosal tumor with fascicular architecture (Hematoxylin Eosin stain, 10×, magnification)—(**C**). Tumor cells express S-100 protein (100×, magnification)—(**D**). The vascular structures of the well-vascularized tumor are marked by smooth muscle actin (SMA), but the tumor cells are negative (100×, magnification).

The laboratory results showed normal values for all tests, including CEA and CA 19-9. Rectal examination revealed no other pathological modifications. Abdominal ultrasonography results showed a swollen gallbladder with wall thickening reaching 3 mm and multiple small gallstones, but no fluid accumulation around the gallbladder. The preoperative routine colonoscopy showed a submucosal intramural growth located at 8 cm from the anal verge, which produced partial luminal stenosis (Figure 2).

Endoscopic biopsy results showed nonspecific spindle-cell proliferation with chronic inflammation, but these findings were not enough to establish a definitive diagnosis. Given the coexistence of symptomatic pelvic organ prolapse and a rectal space-occupying lesion with uncertain nature, a multidisciplinary operative strategy was formulated in conjunction with the gynecologic surgical team. A combined abdominopelvic procedure was planned to address both pathologies.

Genetic testing for hereditary tumor syndromes was not performed, as there was no personal or family history suggestive of inherited cancer predisposition, nor clinical or histopathological features indicating a syndromic association.

Under general anesthesia, a lower midline laparotomy was performed. The surgical examination showed that the gallbladder had swollen, while strong adhesions linked it to the omentum, and a solid nodular growth appeared at the rectosigmoid junction. The examination showed no evidence of peritoneal nodules or hepatic lesions. The gynecologic team performed a Wiart procedure, which involved removing the uterus along with both adnexal structures through a total hysterectomy and bilateral adnexectomy to treat uterine prolapse. The colorectal dissection exposed a solid tumor that grew inside the wall of the rectum through a thin stalk that reached the submucosa without touching the mesorectum or serosa.

**Figure 3:**
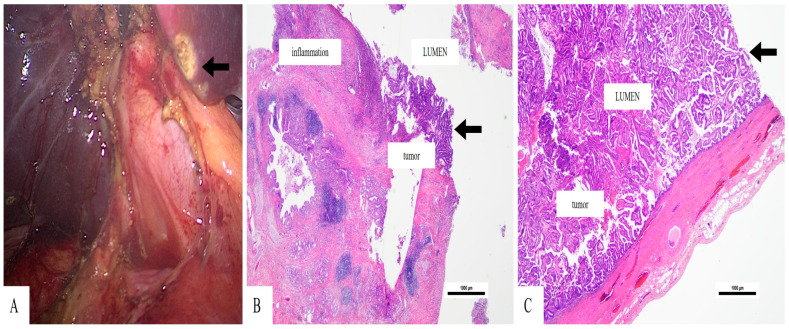
Papillary adenocarcinoma of the gallbladder—(**A**). Intraoperative image of the gallbladder after bipolar dissection, displaying a friable lesion on the fundus (arrow (**A**)—(**B**)). The tumor has a papillary structure, and a rich peri-tumor inflammatory infiltrate can be seen (Hematoxylin Eosin stain, 20×, magnification)—(**C**). The tumor growth is predominantly intraluminal (Hematoxylin Eosin stain, 20×, magnification).

The surgical team performed a rectosigmoid resection with high ligation of the inferior mesenteric vessels before creating a double-stapled termino-terminal colorectal anastomosis, which they placed 6 cm from the anal verge. The surgical team performed a partial mesorectal excision, which surrounded the lesion. Because of the associated biliary pathology, the operative field was extended cranially, and a bipolar cholecystectomy was carried out. The gallbladder contained thick bile and an intraluminal papillary mass in the fundus during dissection, which appeared to be malignant based on visual inspection (Figure 3).

The cystic duct and artery were individually ligated, and the specimen was removed en bloc. The surgical procedure lasted three hours and 40 min of operating time while the team managed bleeding, which remained under 150 mL. The patient received ceftriaxone and metronidazole as prophylactic antibiotics and low-molecular-weight heparin for thromboprophylaxis after surgery.

The rectosigmoid specimen measured 21 cm in length. The tumor appeared as a firm white mass that measured 3.1 × 2.8 × 2.5 cm (length × width × thickness) and extended into the lumen while remaining covered by unbroken mucosa. The microscopic analysis showed a relatively well-defined tumor mass in the submucosa and underlying layers. It consisted of proliferation of spindle cells arranged in intersecting fascicles without necrosis, hemorrhage, or cytological atypia. The tumor cells were immunohistochemically marked by polyclonal S-100 protein (Figure 2). No positivity was seen for CD34, smooth muscle actin (SMA), CD117 (c-kit) or DOG-1. The well-defined intratumorally vascular structures expressed SMA. The Ki-67 proliferative index was below 2%. Based on the microscopic architecture and immunoprofile of the tumor cells, the diagnosis of rectal schwannoma was established. All resection margins were free of tumor.

The gallbladder measured 9 × 3.5 × 3 cm (length × width × thickness) and had a friable papillary exophytic lesion (3.5 cm) that grew from the fundus mucosa. The histological examination revealed proliferation of papillary structures covered by columnar cells, with hyperchromatic nuclei with moderate pleomorphism. The tumor cells were seen in the mucosa, with underlying infiltration to the muscular layer, with extension beyond the serosa, without lymphovascular or perineural invasion (Figure 3). These findings corresponded to a papillary adenocarcinoma of the gallbladder, pT3N0M0 stage (AJCC 8th edition). The surgical margins were free of tumor.

The patient had an uncomplicated recovery following their surgical procedure. The patient started taking oral liquids again on postoperative day two while following a soft food diet starting on day 4. The patient received instructions to begin moving around right away. She did not develop postoperative ileus or experience any complications with her anastomosis. Abdominal drains were removed on day 3. The patient received discharge on postoperative day six because she was in good condition, and normal bowel function was restored with intact anastomosis.

The 2-month follow-up abdominal ultrasonography results showed no signs of disease return or biliary system dilatation. The 6-month contrast-enhanced CT scan showed typical postoperative changes without any sign of cancer relapse in the local or distant body areas. The patient stayed symptom-free during all his scheduled oncologic check-ups, which took place every six months. The patient did not need adjuvant chemotherapy because the tumor was node-negative and had a papillary pattern with an R0 margin. The tumor board, consisting of multiple medical specialties, decided to perform long-term surveillance because the patient had two primary cancers and there was a risk for delayed gallbladder carcinoma recurrence [Table 1].

At her postoperative follow-up visits, the patient reported complete resolution of her initial symptoms and expressed satisfaction with the combined surgical approach. She described a rapid recovery, with restored bowel function and full return to daily activities. The patient appreciated receiving a definitive diagnosis for both lesions and the coordinated care between the surgical and pathology teams.

## 3. Discussion

Synchronous gastrointestinal tumors exist as infrequent medical cases because they contain both benign and malignant tissue types. Their incidence is estimated to be below 0.5% of all digestive neoplasms, and most often the lesions share either a common epithelial origin or arise within contiguous anatomical regions. The present situation stands out because it presents two independent tumors, which include a benign mesenchymal growth (rectal schwannoma) and a dangerous epithelial cancer (gallbladder papillary adenocarcinoma) that doctors discovered accidentally during one pelvic surgical procedure. Our research has not found any previous studies that describe this specific connection.

Schwannomas of the gastrointestinal tract are uncommon, accounting for less than 5% of all mesenchymal tumors [1]. The majority of GISTs occur in the stomach at rates between 60 and 70% followed by the small intestine and colon, but rectal cases make up less than 3% of all reported cases [2,3]. The tumors develop from beneath the mucosa, which makes their identification before surgery difficult because standard imaging and endoscopic biopsy procedures do not generate sufficient tissue for immunohistochemical analysis [4,5].

The histological structure of schwannomas shows spindle cells that form fascicles, which intersect while showing nuclear palisading in Antoni A areas and hypocellular regions in Antoni B. The distinctive feature of these tumors remains their scattered S-100 protein expression together with their absence of CD117, DOG-1, desmin and SMA markers, which differentiates them from gastrointestinal stromal tumors [6]. The patient showed classic schwannoma features through microscopic analysis and immunohistochemical tests, had low cell proliferation and underwent successful complete surgical resection.

Gallbladder carcinoma, although uncommon, is the most frequent malignancy of the biliary tract and typically carries a poor prognosis owing to late diagnosis [7,8]. The spectrum includes papillary adenocarcinoma, which develops from intracholecystic papillary–tubular neoplasms (ICPNs) that function as precursor lesions similar to pancreatic intraductal papillary neoplasms [9]. The lesions show exophytic growth patterns while avoiding direct invasion of the liver, which leads to better treatment outcomes than the typical infiltrating type [10].

Histopathologic identification of papillary growth with perimuscular invasion but intact serosa, as in our patient (pT3N0M0), supports the diagnosis of a localized, potentially curable disease. The preoperative diagnosis of this condition remains difficult because ultrasonography and CT scans produce images that resemble benign gallbladder conditions, including adenomyomatosis and chronic cholecystitis [11]. Surgeons need to stay alert throughout procedures because preoperative imaging results do not show cancer presence, yet papillary structures appear during surgical operations.

The occurrence of two distinct tumors in separate areas of the gastrointestinal system makes it difficult to determine the origin of these tumors. The coincidental theory suggests independent tumor development influenced by age-related genetic instability or cumulative environmental exposure. The field-effect theory suggests that common carcinogenic or mutagenic stimuli trigger neoplasia across different segments of the gastrointestinal tract, which results in multifocal tumors [12].

Research findings show that multiple genetic and molecular pathways exist between tumors, which could explain why patients develop tumors in different organs [13]. Furthermore, chronic inflammatory states—such as cholelithiasis or low-grade mucosal inflammation—can modify the local immune microenvironment and facilitate oncogenic signaling. The occurrence of stochastic coincidence remains possible, although the discovery of two separate cancers in a patient without genetic or syndromic conditions remains scientifically interesting.

Research studies have shown that gallbladder carcinoma presents with similar synchronous symptoms as other primary cancers, including hepatocellular carcinoma and colo-rectal adenocarcinoma, and breast carcinoma [12,13,14]. The complete surgical evaluation during surgery, followed by pathological tissue examination, confirmed no cancer spread in the double primary diagnosis. The research design of our study follows the paradigm because it shows how surgical teams working together can identify extra tumors simultaneously.

Standard imaging tests fail to detect both hidden and small lesions that exist in patients, according to the case. The discovery of unexpected findings during minimally invasive surgery creates difficulties for surgical teams to make intraoperative adjustments while following oncologic principles with their planned surgical approach.

The surgical treatment for schwannoma and papillary adenocarcinoma requires the removal of all tumor tissue while leaving no cancer cells behind. The patient did not need additional adjuvant therapy because the tumor showed no lymphovascular invasion, and the schwannoma was found to be benign. Nevertheless, long-term follow-up remains warranted, particularly for gallbladder carcinoma, given its potential for late recurrence despite apparently favorable histology.

The identification of gallbladder neoplasms from benign mimickers depends on modern imaging technologies, which include high-resolution MRI. The diagnosis of papillary carcinoma becomes more likely when doctors observe intraluminal papillary architecture and no hepatic invasion, and normal fat tissue surrounding the tumor [15]. The preoperative identification of rectal schwannomas becomes possible through MRI and endoscopic ultrasonography, but immunohistochemistry remains the only method to confirm the diagnosis.

The prognosis in this patient is favorable. The medical team successfully removed the benign rectal schwannoma and performed an R0 resection of the gallbladder papillary adenocarcinoma at its early stage. The doctors confirmed her disease-free status during her six-month follow-up appointment. The surveillance program continues to monitor late recurrence and new primary tumors because scientists have not identified the biological elements that lead to simultaneous tumor development.

A limitation of this report is the relatively short follow-up period of six months, which is insufficient to draw conclusions regarding long-term oncologic outcomes, particularly in the context of pT3 gallbladder adenocarcinoma. Nevertheless, the absence of recurrence or metastatic disease during early follow-up supports an initial favorable postoperative course. Continued long-term oncologic surveillance is ongoing.

## 4. Conclusions

The synchronous occurrence of a benign rectal schwannoma and a malignant gallbladder papillary adenocarcinoma represents an exceptionally rare clinical finding. This case underscores the importance of comprehensive intra-abdominal exploration, meticulous histopathologic evaluation, and multidisciplinary decision-making in patients presenting with complex or incidental lesions. Although the coexistence of histologically distinct tumors may be coincidental, shared molecular or immunologic mechanisms cannot be excluded. Complete surgical excision with negative margins remains the cornerstone of management, and long-term surveillance is essential to detect recurrence or additional neoplastic processes. Reporting such unusual tumor associations contributes valuable insight into the spectrum of dual tumor pathogenesis within the gastrointestinal tract.

## Figures and Tables

**Table 1:** Treatment Timeline.

Date/Interval	Event	Details
Preoperative period	Initial presentation	Stage IV uterovaginal prolapse, constipation; rectal discomfort
Preoperative workup	Imaging & colonoscopy	Rectal submucosal mass; gallstones; normal labs
Day 0	Combined surgery	Wiart procedure + rectosigmoid resection + bipolar cholecystectomy
Post-op day 6	Hospital discharge	Full recovery; normal bowel function
2-month follow-up	Abdominal US	No recurrence; no biliary dilation
6-month follow-up	Contrast-enhanced CT	No evidence of disease recurrence

## Data Availability

The data that support the findings of this study are available from the main author, C.C., upon request, catalin.cosma@umfst.ro.

## Abbreviations

AJCC	American Joint Committee on Cancer
CK	Cytokeratin
CT	Computed Tomography
DOG-1	Discovered on GIST-1
GIST	Gastrointestinal Stromal Tumor
H&E	Hematoxylin and Eosin
HPF	High-Power Field
ICPN	Intracholecystic Papillary–Tubular Neoplasm
IHC	Immunohistochemistry
MRI	Magnetic Resonance Imaging
R0	Complete Tumor Resection with Negative Margins
SMA	Smooth Muscle Actin
S-100	Schwann Cell Marker Protein S-100
US	Ultrasonography
Wiart	Wiart Procedure (Total Hysterectomy with Bilateral Adnexectomy for Genital Prolapse)

## References

[1] Bohlok A., El Khoury M., Bormans A., Galdon M.G., Vouche M., El Nakadi I., Donckier V., Liberale G. (2018). Schwannoma of the colon and rectum: A systematic literature review. World J. Surg. Oncol..

[2] Qi Z., Yang N., Pi M., Yu W. (2021). Current status of the diagnosis and treatment of gastrointestinal schwannoma (Review). Oncol. Lett..

[3] Zippi M., Pica R., Scialpi R., Cassieri C., Avallone E.V., Occhigrossi G. (2013). Schwannoma of the rectum: A case report and literature review. World J. Clin. Cases.

[4] Zhang Y.-J., Yuan M.-X., Wen W., Jian Y., Zhang C.-M., Yuan J., He L. (2025). Endoscopic full-thickness resection of rectal schwannoma: A case report. World J. Gastrointest. Endosc..

[5] Zhang K., Qu S., Li J., Cheng Y., Shi J., Liu T. (2018). A case report of rectal schwannoma treated with laparoscopic proctectomy. Medicine.

[6] Vohra R.R., Bokhari S.F.H., Owais M., Haseeb M., Kharal F. (2022). Schwannoma of the ascending colon in a 22-year-old male: A case report. Cureus.

[7] Albores-Saavedra J., Tuck M., McLaren B.K., Carrick K.S., Henson D.E. (2005). Papillary carcinomas of the gallbladder: Analysis of noninvasive and invasive types. Arch. Pathol. Lab. Med..

[8] Wan X., Zhang H., Chen C., Yang X., Wang A., Zhu C., Fu L., Miao R., He L., Yang H. (2014). Clinicopathological features of gallbladder papillary adenocarcinoma. Medicine.

[9] Adsay V., Jang K.-T., Roa J.C., Dursun N., Ohike N., Bagci P., Basturk O., Goodman M., Kooby D., Maithel S.K. (2012). Intracholecystic papillary-tubular neoplasms (ICPN) of the gallbladder: Clinicopathologic and immunohistochemical analysis of 123 cases. Am. J. Surg. Pathol..

[10] Henson D.E., Albores-Saavedra J., Code D. (1992). Carcinoma of the gallbladder: Histologic types, stage of disease, grade, and survival rates. Cancer.

[11] Araki T., Hihara T., Karikomi M., Kachi K., Uchiyama G. (1988). Intraluminal papillary carcinoma of the gallbladder: Prognostic value of computed tomography and sonography. Gastrointest. Radiol..

[12] Dash S., Samantara S.K., Pani K.C., Ranjit M. (2023). An unusual case series of synchronous primary malignancies: Carcinoma gallbladder with renal cell carcinoma, carcinoma gallbladder with carcinoma colon, carcinoma gallbladder with carcinoma breast. J. Cancer Res. Ther..

[13] Xu Y., Chen Q.-N., Wang H., Liu N.-B., Shi B.-M. (2020). Synchronous hepatocellular carcinoma and gallbladder adenocarcinoma with neuroendocrine differentiation: A case report and literature review. BMC Surg..

[14] Choi J., Kim H.J., Jang S.K., Paik S.Y., Kim K.H. (2020). Synchronous cancers of hepatic angiosarcoma and gallbladder adenocarcinoma, mimicking gallbladder cancer with hepatic invasion: A case report. Investig. Magn. Reson. Imaging.

[15] Vendrami C.L., Magnetta M.J., Mittal P.K., Moreno C.C., Miller F.H. (2021). Gallbladder carcinoma and its differential diagnosis at MRI: What radiologists should know. RadioGraphics.

